# Selenite Reduction and the Biogenesis of Selenium Nanoparticles by *Alcaligenes* *faecalis* Se03 Isolated from the Gut of *Monochamus alternatus* (Coleoptera: Cerambycidae)

**DOI:** 10.3390/ijms19092799

**Published:** 2018-09-17

**Authors:** Yuting Wang, Xian Shu, Qing Zhou, Tao Fan, Taichu Wang, Xue Chen, Minghao Li, Yuhan Ma, Jun Ni, Jinyan Hou, Weiwei Zhao, Ruixue Li, Shengwei Huang, Lifang Wu

**Affiliations:** 1 Key Laboratory of High Magnetic Field and Ion Beam Physical Biology, Hefei Institutes of Physical Science, Chinese Academy of Sciences, Hefei 230031, China; wangyuting2006@outlook.com (Y.W.); sx360775419@gmail.com (X.S.); USTCzq1@outlook.com (Q.Z.); chenxueahau@163.com (X.C.); limh@ipp.ac.cn (M.L.); yuhanmie@ipp.ac.cn (Y.M.); nijun@ipp.ac.cn (J.N.); jyhou@ipp.ac.cn (J.H.); annyzhao@ipp.ac.cn (W.Z.); 2 School of Life Sciences, University of Science and Technology of China, Hefei 230026, China; 3 The Sericultural Research Institute, Anhui Academy of Agricultural Science, Hefei 230061, China; fantao1116@sohu.com (T.F.); wangtaichu@eyou.com (T.W.); li-ruixue@163.com (R.L.); 4 Key Laboratory of Environmental Toxicology and Pollution Control Technology of Anhui Province, Hefei Institutes of Physical Science, Chinese Academy of Sciences, Hefei 230031, China

**Keywords:** selenite reduction, *Alcaligenes faecalis*, biogenic selenium nanoparticles, electron microscopy analysis, real-time PCR

## Abstract

In this study, a bacterial strain exhibiting high selenite (Na_2_SeO_3_) tolerance and reduction capacity was isolated from the gut of *Monochamus alternatus* larvae and identified as *Alcaligenes faecalis* Se03. The isolate exhibited extreme tolerance to selenite (up to 120 mM) when grown aerobically. In the liquid culture medium, it was capable of reducing nearly 100% of 1.0 and 5.0 mM Na_2_SeO_3_ within 24 and 42 h, respectively, leading to the formation of selenium nanoparticles (SeNPs). Electron microscopy and energy dispersive X-ray analysis demonstrated that *A. faecalis* Se03 produced spherical electron-dense SeNPs with an average hydrodynamic diameter of 273.8 ± 16.9 nm, localized mainly in the extracellular space. In vitro selenite reduction activity and real-time PCR indicated that proteins such as sulfite reductase and thioredoxin reductase present in the cytoplasm were likely to be involved in selenite reduction and the SeNPs synthesis process in the presence of NADPH or NADH as electron donors. Finally, using Fourier-transform infrared spectrometry, protein and lipid residues were detected on the surface of the biogenic SeNPs. Based on these observations, *A. faecalis* Se03 has the potential to be an eco-friendly candidate for the bioremediation of selenium-contaminated soil/water and a bacterial catalyst for the biogenesis of SeNPs.

## 1. Introduction

Selenium (Se) is a naturally occurring chalcogen element that possesses both metallic and nonmetallic properties [1]. It is an essential micronutrient for both prokaryotes and eukaryotes at low concentrations, but it can be toxic to organisms at higher levels [2]. For example, Se can act as an antioxidant and protect against the cellular damage caused by oxygen radicals; however, an overdose of Se can disrupt the integrity of proteins and decrease cellular enzymatic activity, resulting in chronic or acute selenosis [3,4]. Se exists in nature in multiple organic and inorganic forms, two of which there are mobile, water-soluble, bioavailable and toxic: selenite (SeO_3_^2−^) and selenate (SeO_4_^2−^). While selenite is generally more toxic than selenate, both can be easily absorbed by plants and animals from Se-rich soil or water containing Se, thus entering the food chain and posing a potential threat to animals and humans [5,6]. Thus, Se is of significant research interest in terms of environmental protection and public health.

Compared with selenite and selenate, elemental selenium (Se^0^) is insoluble and shows little or no toxicity in terrestrial and aquatic environments. Therefore, the reduction of both selenate and selenite to Se^0^ has been identified as an ideal strategy for selenium detoxification and Se recovery in contaminated water, soil, and industrial effluent [7]. While various physicochemical and biological methods have been applied to reduce selenate and selenite into Se^0^ [8,9,10], biological approaches are generally preferred due to additional benefits such as their low cost, eco-friendly nature, and ability to employ self-generating catalysts [4]. Microorganisms thus play a crucial role in the transformation of selenite and selenate via metabolic reactions, and a variety of microorganisms, including *Duganella* sp. [3], *Agrobacterium*. sp. [3], *Enterobacter cloacae* [11], *Vibrio natriegens* [12], *Pseudomonas putida* [13], *Bacillus cereus* [14], *Bacillus mycoides* [15], and *Shewanella oneidensis* [16], have been reported to have reducing ability. For example, Yee et al. [11] reported that Se(VI) reduction and the precipitation of Se^0^ by facultative anaerobes *E. cloacaea* are regulated by oxygen-sensing transcription factors (such as fumarate nitrate reduction regulators) and occur under suboxic conditions. Avendaño et al. [13] found that *P. putida* KT2440 is able to reduce selenite (but not selenate) aerobically to Se nanoparticles (SeNPs), with the synthesized SeNPs located in the surrounding medium or bound to the cell membrane. In addition, research on the accumulation and metabolism of selenium in *Saccharomyces cerevisiae* also showed that yeast cells can convert selenite and selenate to selenoamino acids, in particular selenomethionine, and can accumulate up to 3000 μg·g ^−1^ of selenium [17]. The presence of Se^0^ in yeast cell structures was also reported by Jiménez-Lamana et al. [18].

It is interesting to note that a number of these microbes can biosynthesize SeNPs of a defined size and shape in the selenite/selenate reduction process [11,13,19]. Compared to physicochemical methods, the biosynthesis of SeNPs has several advantages, such as specificity, safety, stability, and an eco-friendly nature [20], in addition to specific spectral and optical properties [21,22] and antimicrobial and anticancer activity [23,24,25]. Because of this, the microbial biosynthesis of SeNPs has great potential for use in selenium bioremediation and nanobiotechnology.

In this work, we studied the reduction of selenite and the biosynthesis of SeNPs by the bacterium *Alcaligenes faecalis* Se03, which was isolated from the gut of the larvae of the herbivorous insect *Monochamus alternatus* (Coleoptera: Cerambycidae). *A. faecalis* is an aerobic β-proteobacterium commonly found in the environment that exhibits heterotrophic nitrification and aerobic denitrification abilities [26]. Genome mining in *A. faecalis* has also demonstrated that its central metabolism is equipped with enzymes necessary to produce a high yield of reducing power (i.e., NAD(P)H equivalents) [27]. This ability is useful for selenite bioremediation, meaning *A. faecalis* is an attractive bacterial candidate for applications related to this biocatalytic process.

## 2. Results and Discussion

### 2.1. Isolation, Characterization, and Identification of Bacterial Strains

**Figure 1:**
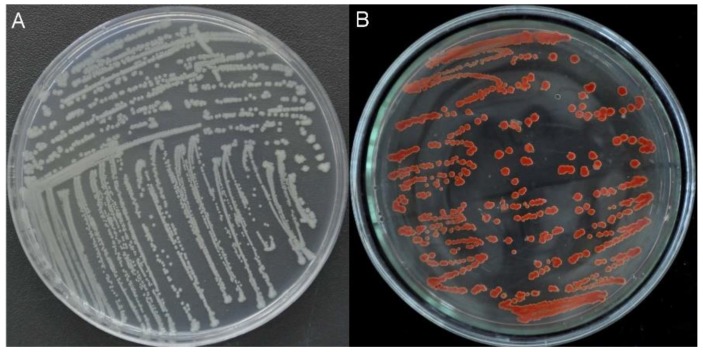
Growth of strain Se03 on YEP (Yeast Extract Peptone) agar plates in absence (**A**) and presence (**B**) of 5.0 mM selenite. The red colony color indicates selenite reduction and the formation of elemental selenium (Se^0^).

In previous research, several species of selenite-reducing bacteria, such as *Streptomyces* sp. [28], *Stenotrophomonas maltophilia* [29], and *Burkholderia fungorum* [10], have been isolated from the soil or rhizosphere. However, few bacterial species have been isolated from insects. Insects harbor large gut communities of specialized bacteria because their gut presents a unique environment for microbial colonization [30]. In this study, 13 bacterial strains were isolated from gut samples of *M. alternatus* using yeast extract peptone (YEP) plates supplemented with 10 mM sodium selenite, suggesting that the insect gut could be a new source of microorganisms capable of transforming selenite into elemental selenium (Se^0^). Of the 13 strains, isolate #03 exhibited good growth and the ability to reduce selenite to red Se^0^ (Figure 1). Therefore, this isolate (named Se03) was chosen for further study.

**Figure 2:**
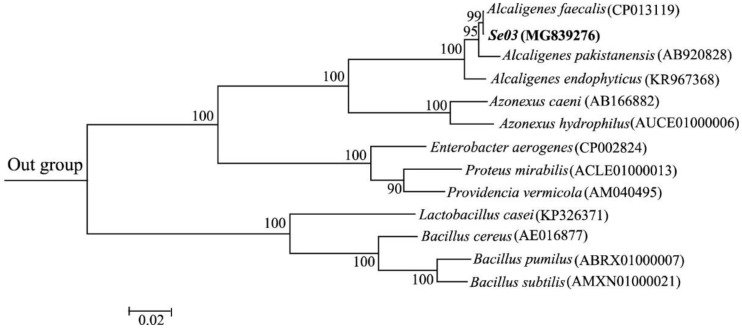
Maximum likelihood tree based on the 16S rRNA gene sequence of isolate Se03 and the sequences of representative strains from GenBank. The scale bars indicate 0.02 substitutions per site. Sphingobacterium zeae (KU201960) was used as an outgroup.

Based on 16S rRNA gene sequence and phylogenetic evolution analysis, isolate Se03 showed a high degree of similarity (99%) with *Alcaligenes faecalis* (Figure 2). Given that it also exhibited the typical biochemical and physiological characteristics of *A. faecalis* (Table 1), the strain Se03 was identified as *A. faecalis* Se03. *A. faecalis* is a Gram-negative, rod-shaped bacterium that is commonly found in soil, water, and environments associated with animals. In addition, it has been recently demonstrated that *A. faecalis* can degrade a wide range of aromatic compounds including polyaromatic hydrocarbon [27], naphthalene, and phenanthrene [31], and can detoxify heavy metals [32,33]. However, this is the first study to demonstrate that an *A. faecalis* strain can reduce selenite to Se^0^ and biosynthesize SeNPs.

### 2.2. Selenite Reduction and the Formation of Elemental Selenium

To determine the selenite tolerance of the Se03 isolate, bacterial cells were grown in YEP medium with different concentrations of selenite (0–300 mM). Minimum inhibitory concentration (MIC) assays demonstrated that strain Se03 had an extremely high tolerance to selenite; it was able to grow and produce Se^0^ in the presence of selenite concentrations of up to 120 mM. This indicates that *A. faecalis* Se03 has a higher level of resistance to selenite than that reported for some other bacteria such as *Burkholderia fungorum* (5 mM) [10] and *Azoarcus* sp. CIB (8 mM) [19], and it is similar to other highly selenite-tolerant strains, including *Vibrio natriegens* (100 mM) [12] and *Pseudomonas moraviensis* (120 mM) [34].

**Figure 3:**
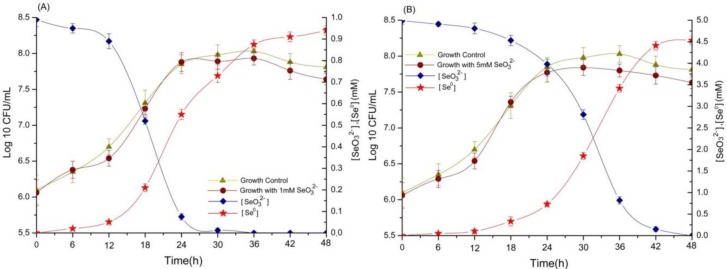
Time courses of bacterial growth, SeO_3_^2−^ reduction, and Se^0^ formation by the strain *A. faecalis* Se03 grown in YEP medium supplied with (**A**) 1 mM Na_2_SeO_3_, and (**B**) 5.0 mM Na_2_SeO_3_. Each test was performed in triplicate and error bars represent the standard deviation.

The selenite reduction ability of *A. faecalis* Se03 was evaluated using liquid YEP medium supplemented with 1.0 and 5.0 mM Na_2_SeO_3_ (Figure 3). The Shapiro-Wilk test showed a normal distribution for all selenite reduction and Se^0^ formation data (Supplementary Table S1). Depletion of SeO_3_^2−^ was observed within 6 hours at SeO_3_^2−^ concentrations of 1.0 and 5.0 mM, though only 5.0% and 1.8% of the SeO_3_^2−^ had been reduced at this stage, respectively. SeO_3_^2−^ reduction and Se^0^ formation by Se03 appeared to be a continuous process that was associated with this strain’s growth kinetics. In the bacterial cultures with an initial selenite concentration of 1.0 mM, most of the selenite (>90%) was exhausted during exponential growth phase (between 12 and 24 h) and then completely reduced after 36 h of incubation (Figure 3A). In contrast, the reduction behavior of *A. faecalis* Se03 changed when cultured in a YEP medium with 5 mM selenite. Selenite reduction occurred at the very end of the exponential growth phase and well into the stationary phase. During the exponential phase, only 10% of the initial selenite was reduced, while the remaining selenite was depleted during the stationary phase (Figure 3B). These findings are similar to those of Lampis et al. [29], who showed that SeO_3_^2−^ reduction and Se^0^ formation were associated with the growth kinetics of *S. maltophilia* SeITE02. When selenite was added to the culture medium at a low concentration (0.5 mM), it was 100% depleted within an incubation period of 52 h (i.e., during the exponential phase). However, when supplemented with 2.0 mM selenite, less than 10% of the SeO_3_^2−^ had been reduced after 52 h of incubation, while 86% of the initial selenite was reduced after 192 h of incubation (i.e., during the stationary phase). A similar pattern was also discovered by Khoei et al. [10], who found that about 75% of initial SeO_3_^2−^ depletion occurred during the exponential growth phase when *Burkholderia fungorum* 95 was cultured with 0.5 mM selenite. However, in the presence of 2.0 mM SeO_3_^2−^, only 10% of the SeO_3_^2−^ was depleted during the first 24 h, with the majority of the reduction occurring during the stationary growth phase (between 24 and 48 h). In addition to this, it has been reported that *Bacillus mycoides* SeITE01 is able to reduce most of the SeO_3_^2−^ during the exponential phase at a SeO_3_^2−^ concentration of 0.5 mM. However, at a concentration of 2 mM, only 25% of the SeO_3_^2−^ was reduced during the exponential growth stage, with the remaining SeO_3_^2−^ depleted during the stationary phase [15]. Therefore, it seems clear that, for *A. faecalis* Se03, the selenite reduction rate and efficiency are most likely related to the initial selenite concentration and/or the bacterial growth phase. It is also possible that selenite reduction is mediated by cellular reductases and/or reducing compounds whose production and consumption are linked to the growth phase of the microbe [29].

Furthermore, it is quite generally believed that elemental selenium is endowed with a characteristic red color. In this study, the reduction and consequent depletion of SeO_3_^2−^ led to the culture medium turning bright red, indicating the presence of Se^0^, which is generally associated with this color. No color change was observed in the two control flasks, one containing only selenite and the other containing only the bacterial strain, suggesting that *A. faecalis* Se03 plays a role in selenite reduction and Se^0^ formation. About 90% of the reduced selenite was transformed into Se^0^ at both concentrations tested, although the SeO_3_^2−^ concentration of 5.0 mM required a significantly longer period of time (Figure 3B). It should be noted that a delay in the formation of Se^0^ following SeO_3_^2−^ reduction was observed, with the bacterial cultures turning light red after 12 and 18 h of incubation at concentrations of 1.0 and 5.0 mM SeO_3_^2−^, respectively (Figure 3). These results are in good agreement with the Se^0^ levels in bacterial cultures measured using a spectrophotometer in this study. For example, at a concentration of 1.0 mM SeO_3_^2−^, 11% of the initial selenite was reduced after 12 h of incubation, but only 5.1% was transformed into detectable Se^0^. The delay in the formation of Se^0^ has also been reported by Lampis et al. [15] and Fernández-Llamosas et al. [12]. It is assumed to be caused by the formation of metabolic intermediates such as RSeR prior to Se^0^ formation [29,35]. However, past research on SeNPs synthesized via microorganism activity has mostly focused on bacteria or fungi, while Se^0^ has rarely been detected and quantified in the presence of yeast. Kieliszek et al. [17] reported that yeast cells have the ability to reduce selenite(IV) ions to Se^0^ in the presence of sulphate(IV) ions. Jiménez-lamana et al. [18] also verified the presence of inorganic nanoparticulate selenium in Se-rich yeast *Saccharomyces cerevisiae* firstly by using single particle inductively coupled plasma mass spectrometry (ICPMS) and suggested that this species should be included in the speciation scheme for this element in Se-rich yeasts.

### 2.3. Location of SeNPs within Cell Cultures of A. faecalis Se03

**Figure 4:**
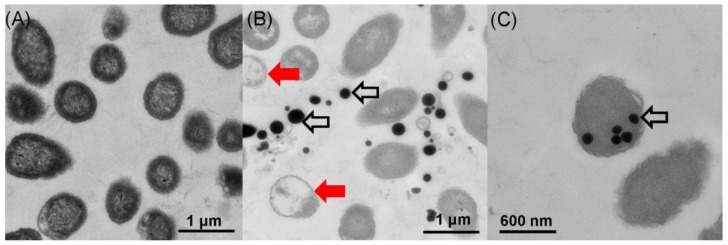
TEM images of *A. faecalis* Se03 in (**A**) the absence and (**B**,**C**) presence of 5mM selenite after 36 h incubation. Electron-dense nanoparticles (white arrows) located extracellularly (**B**) or intracellularly (**C**). Red arrows indicate “empty ghost” cells.

**Figure 5:**
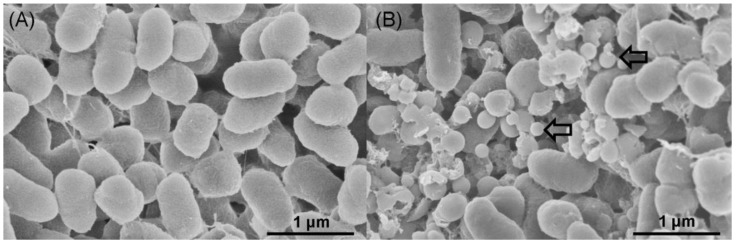
SEM micrographs of *A. faecalis* Se03 cultures grown in (**A**) absence and (**B**) presence of 5.0 mM selenite after 36h incubation. Electron-dense nanoparticles (white arrows) located extracellularly.

Transmission electron microscopy (TEM) and scanning electron microscopy (SEM) were used to determine the location of SeNPs produced by *A. faecalis* Se03. TEM analysis found that most of the electron-dense nanoparticles were located in the extracellular spaces and only a few were observed in the cytoplasm (Figure 4B,C), while these particles were not detected in cell cultures in the absence of SeO_3_^2−^ (Figure 4A). The particles were spherical and of various sizes (i.e., diameters ranging from approximately 100 to 400 nm), similar to those found in in *Duganella* sp. [3], *Agrobacterium* sp. [3], *E. cloaocae* [11], *B. selenitireducens* [20]. SEM micrographs also showed the extracellular location of the electron-dense nanoparticles, their spherical shape, and various sizes (Figure 5, indicated by white arrows). Interestingly, empty ghost cells were observed using TEM (indicated by the red arrows in Figure 4B). Empty ghost cells were also found in a study of *Candida utilis* ATCC 9950. These ghost cells were observed in aging yeast as a result of cell membrane breakage and cytoplasm leakage when *C utilis* ATCC 9950 was cultured in media supplemented with sodium selenite [36]. Lampis et al. [29] also found that empty cells were abundant during the stationary growth phase of *S. maltophilia* SeITE02 in the presence of 0.5 mM selenite, possibly due to the release of Se^0^ particles into the medium. However, there is a lack of experimental evidence to determine whether vesicular secretion or cell lysis is involved in the release of intracellularly formed Se^0^ nanoparticles by *A. faecalis* Se03. More research is thus needed to further elucidate the release mechanisms of Se^0^ nanoparticles.

### 2.4. Characterization of SeNPs Produced by A. faecalis Se03

#### 2.4.1. DLS Analyses and SEM-EDX

**Figure 6:**
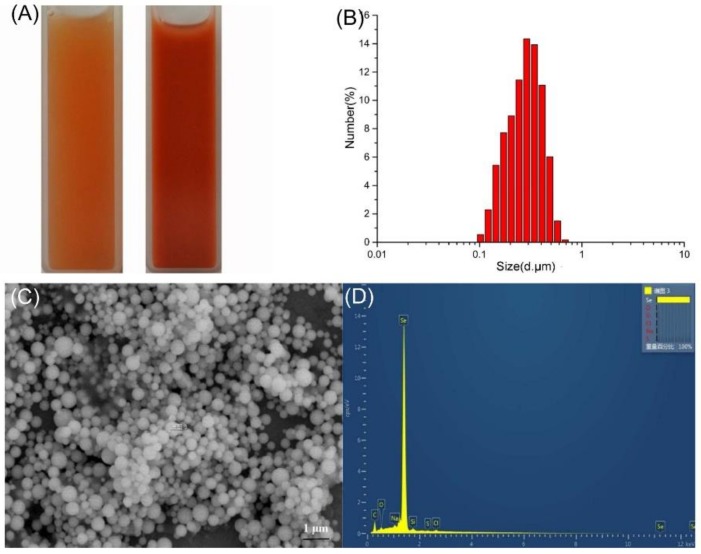
DLS spectra of purified SeNPs produced by *A. faecalis* Se03 in YEP supplemented with 5.0 mM selenite. (**A**) Selenite-dosing cells (left) and purified nano-selenium (right); (**B**) size distribution of purified SeNPs; (**C**) SEM micrographs of purified SeNPs. (**D**) EDX analysis of purified SeNPs showing its selenium composition.

Results of the dynamic light scattering (DLS) analysis of the purified SeNPs are presented in Figure 6. It was revealed that the average diameter of the nanoparticles recovered after 36 h of incubation was 273.8 ± 16.9 nm, similar to that found in *Pseudomonas putida* (266 nm) [13], and *Bacillus selenitireducens* (200 nm) [37]. Bioproduced SeNPs of various sizes have been described in *Vibrio natriegens* (136 ± 31 nm) [12], *Azoarcus* sp. CIB (174 ± 36 nm in aerobic cultures and 90 ± 26 nm in anaerobic cultures) [18], and *Bacillus mycoides* SeITE01 (50 to 400 nm). Energy-dispersive X-ray (EDX) analysis showed that the purified SeNPs were spherical and clearly indicated the presence of selenium, with specific absorption peaks observed at 1.37, 11.22, and 12.49 keV.

#### 2.4.2. The Fourier transform infrared spectroscopy (FTIR) Analysis

**Figure 7:**
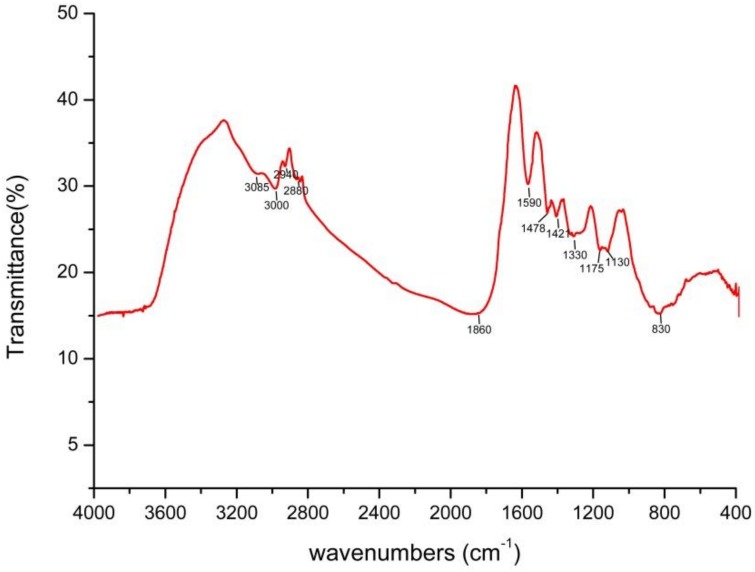
The FTIR spectrum of Bio-SeNPs registered in the 4000–400 cm^−1^ infrared regions.

The Fourier transform infrared spectroscopy (FTIR) spectrum of the SeNPs is presented in Figure 7. Stretching bands can be observed at 3085 (NH stretching in proteins and peptides), 3000 (the C-H broad alkyl stretching band), 2940 (asymmetric stretching of C-H bonds in the methyl group of both aliphatic and aromatic compounds), 2880 (fatty acyl chains), 1860 (the symmetric stretching mode of anhydrides), 1590 (C=O vibrations), 1478 (symmetric NH_3_^+^-deformation vibrations), 1421 (symmetric COO-stretching vibrations), 1330 (lipid content, C-H band vibrations, or syringyl ring breathing with C=O stretching), 1175 (C=O stretching vibrations), 1130 (C-N stretching vibrations of phenols), and 840 (the aromatic ring group). Thus, the FTIR analysis clearly demonstrated that organic residues such as carbohydrates, lipids, and proteins were present on the surface of the SeNPs produced by *A. faecalis* Se03. Previous studies on *Thauera selenatis* [38] and *S. maltophilia* SeITE02 [29] have identified the functional groups of the bacterial biomolecules involved in the selenite reduction and the stabilization (capping) process for synthesized SeNPs. Therefore, our findings not only indicate bacterial protein-mediated selenite reduction but also corroborate the synthesis and stabilization of SeNPs by the proteins present in the bacteria [38].

### 2.5. Biocatalytic Selenite Reduction Activity Assays

**Figure 8:**
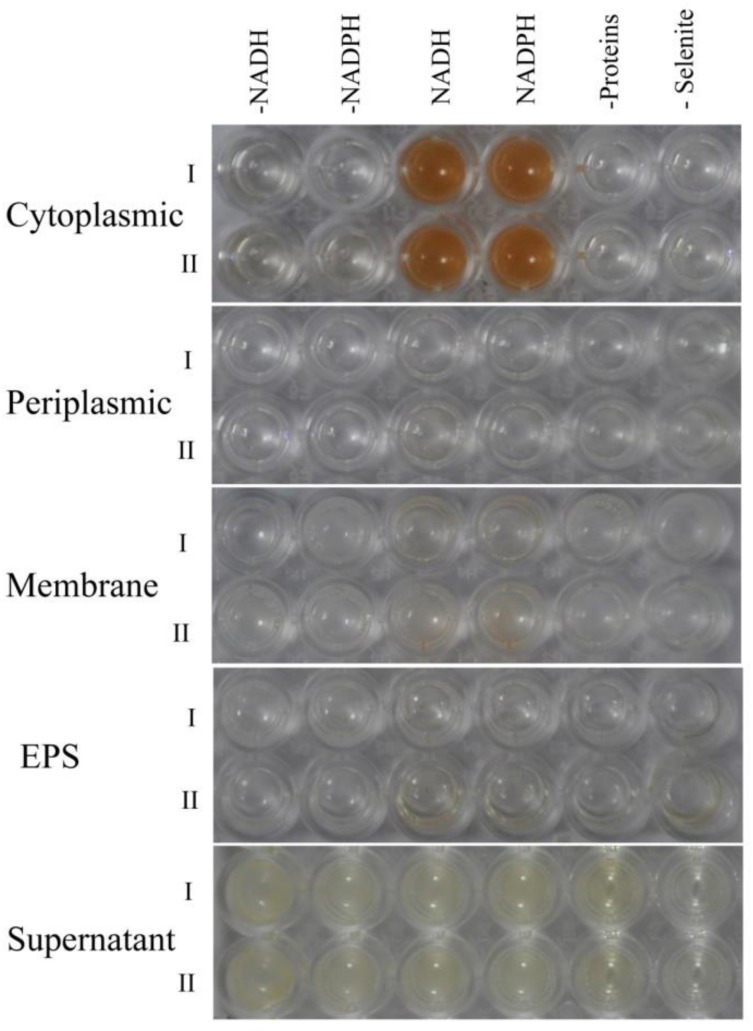
Selenite reduction assay on different subcellular fractions (cytoplasmic, periplasmic, and membrane), supernatant, and exopolysaccharide. All experiments were performed in duplicate (indicated by roman numbers), with addition of 5.0 mM SeO_3_^2−^ and 2.0 mM NADH or NADPH. While 3 following control negatives were performed: without protein fractions or supernatant or EPS, without selenite, without NADH or NADPH.

To determine the mechanism of selenite reduction to Bio-SeNPs in *A. faecalis* Se03, the selenite reduction activity of different fractions of the cell culture were tested. Results presented in Figure 8 clearly demonstrated that selenite reduction activity in Se03 cells is localized in the cytoplasmic fraction. Furthermore, selenite reduction by the cytoplasmic fraction is an enzymatic process since an electron donor (NADH or NADPH) is required for this reaction to take place. The observed involvement of enzymes in the reduction of selenite was also found in other microorganisms. Khoei et al. [10] found that cytoplasmic fractions of two *Burkholderia fungorum* strains (*B. fungorum* DBT1 and *B. fungorum* 95) exhibited selenite reduction activity in the presence of an electron donor (NADH or NADPH) while supernatant, exopolysaccharide (EPS) or membrane fraction possessed no activity for both strains. In addition, Lampis et al. [28] found that the selenite reduction activity of *S. maltophilia* SeITE02 cells was mainly attributed to the cytoplasmic fraction with NADH served as an electron donor. Thus, it is possible to infer that selenite that imported into the *A. faecalis* Se03 cell was reduced and formed inside as small Se^0^ seeds in the cytoplasm by thioredoxin reductase [39], or NADH/NADPH related reductases [40]. Interestingly, selenium nanoparticles accumulated extracellularly to a noticeable extent after 36 h incubation as shown in TEM micrograph (Figure 4B), suggesting the SeNPs formed inside the cell could be released into the extracellular space through certain export mechanism. Several systems exist in Gram-negative bacteria for SeNPs secretion out of the cell including outer membrane vesiculation [41], membrane-associated efflux pumps [42]. In addition, cell lysis prior to secretion is also possible way out for Se^0^ particles formed inside the cytoplasm [43]. Despite vesicles produced by Gram-negative bacteria can expulse material located in the periplasmic space or cytoplasm, such vesicles are about 20–250 nm as suggested by Kulp and Kuehn [41]. However, most of the observed Se^0^ spheres (about 60%) were larger than that size (Figure 6B), while Se nanoparticles were too large to be released from bacterial cells without rupturing the cell walls. More importantly, empty ghost cells as a symbol of cell membrane breaking and cytoplasm leakage were observed by TEM (indicated by red arrows in Figure 4B). Thus, the majority of extracellularly observed Se^0^ spheres were most likely intracellularly produced and subsequently released following cell lysis, as reported in other microorganisms such as anaerobic granular sludge [44], *Duganella* sp. and *Agrobacterium* sp. [3] and *Desulfovibrio desulfuricans* [45]. However, additional experiments should be performed to address both the nature of Se^0^ formation and the release mechanism of SeNPs produced by *A. faecalis* Se03.

### 2.6. Real-time PCR Analysis

**Figure 9:**
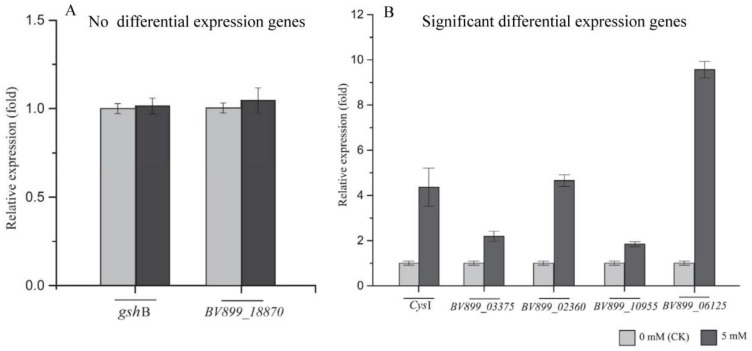
Real-time PCR results of expression levels of selected genes (**A**) Not significant differentially expressed genes, (**B**) significant differential expression of genes. Data are shown as fold changes by calculating expression levels of selenite treated samples compared to untreated (defined as 1). Data represent an average of three biological replicates ± SD.

Previous studies have found that selenite can react with glutathione (GSH) to form Se^0^ and reduced GSH through Painter-type reactions [46] and that other enzymatic systems, such as sulfite reductase, flavoprotein, and thioredoxin, are also involved in the reduction of selenite and Se^0^ formation [40,47]. Therefore, the mRNA expression of genes suspected to be involved in selenite reduction and SeNP formation in *A. faecalis* Se03, including glutathione synthetase (*gsh*B), sulfite reductase (*Cys*I), the sulfate transporter subunit (*BV899_03375*), flavoprotein sulfite reductase (*BV899_18870*), thioredoxin reductase (*BV899_02360*), peroxiredoxin (*BV899_10955*), and superoxide dismutase (*BV899_06125*), was assessed using real-time PCR (Table 2). The Shapiro-Wilk test showed a normal distribution of expression fold changes for all selected genes (Supplementary Table S1). As shown in Figure 9, the mRNA expression of *gsh*B and *BV899_18870* in cells cultured with selenite was not different from cells without selenite treatment, suggesting that GSH might not be involved in the reduction of selenite to SeNPs. However, selenite treatment significantly promoted the mRNA expression of *Cys*I, *BV899_03375*, *BV899_02360, BV899_10955*, and *BV899_06125* (a 4.4-, 2.2-, 4.7, 1.8-, and 9.6-fold increase, respectively).

Because the chemical and physical characteristics of S and Se are very similar to each other, the enzymes involved in sulphate metabolism and in the trans-sulphuration pathway typically do not discriminate between S and Se compounds [48]. Thus, selenate and selenite could be transported into cells via the sulfate transport system and reduced to Se^0^ in the cytoplasm [49,50]. Some bacteria are able to utilize selenite and selenate in their respiratory chain as electron acceptors, often along with sulphites and sulphates [47]. Harrison et al. [51] previously reported that the expression of sulfite reductase in *Clostridium pasteurianum* was induced by the presence of selenite and that it participated in selenite reduction in cells, while Kieliszek et al. [17] suggested that selenite(IV) reduction in yeast cells can be catalyzed by sulfate reductase using NADPH as a reducing agent. Furthermore, the thioredoxin system consisting of NADPH, thioredoxin reductase, and thioredoxin can reduce protein disulfides via redox-active dithiols, and it has been reported to be active in the in vitro reduction of selenite in *Bacillus subtilis* [52] and *Pseudomonas seleniipraecipitans* [1]. In addition, enzymes that are capable of scavenging reactive oxygen species (ROS), including peroxiredoxins and superoxide dismutase therefore, have been found to participate in the process of selenite reduction and Se^0^ formation and are induced by both selenate and selenite treatment under aerobic conditions [53,54].

Overall, this data suggests that *A. faecalis* Se03 may reduce selenite and produce Se^0^ via the action of reductases, such as sulfite reductase and thioredoxin reductase, instead of via GSH-mediated Painter-type reactions. However, it is likely that multiple mechanisms are involved in the reduction of selenite in the *A. faecalis* Se03 strain. Therefore, additional research based on proteomic analysis and mutant forms is necessary to identify the possible reduction mechanisms in *A. faecalis* Se03.

## 3. Materials and Methods

### 3.1. Chemicals and Culture Medium

YEP broth was purchased from Qingdao Hopebio-Technology (Qingdao, China). Sodium selenite (Na_2_SeO_3_, ≥99%) was purchased from Sigma-Aldrich (St Louis, MO, USA) and diluted with distilled deionized water (ddH_2_O) to prepare a Na_2_SeO_3_ stock solution (2 mol/L) and filter-sterilized. Aliquots of Na_2_SeO_3_ from the filter-sterilized stock solution were added to the YEP growth media when needed. All other analytical grade reagents were purchased from Sangon Biotech (Shanghai, China) and Sinopharm Chemical Reagent (Shanghai, China).

### 3.2. Bacterial Isolation and Identification

The third larval stage of the herbivorous insect *M. alternatus* (Coleoptera: Cerambycidae) was collected from *Pinus massoniana* growing in the seleniferous soils of Nanyang Forest Farm, Chizhou, Anhui Province, China. After returning to the laboratory, the larvae were dissected immediately and the gut extracts were pooled and homogenized as described by Huang et al. [55]. Briefly, 1 gram of a homogenized gut sample was serially diluted with sterilized distilled water by a factor of 10^1^–10^9^. Aliquots (100 μL) of each dilution were spread on YEP plates containing 10 mM sodium selenite and incubated for 2–3 days at 30 °C until visible colonies appeared on the plates. A red colony indicated selenite reduction. Individual colonies were streaked on new media to obtain pure bacterial monocultures. Of these monocultures, isolate #03 (named Se03) was chosen for this study based on its apparent rate of growth and ability to reduce selenite to red elemental Se (Se^0^).

The biochemical and physiological analysis of the Se03 isolate was conducted according to standard methods [56]. The 16S rRNA gene sequence of the Se03 isolate was amplified using bacterial universal primer 27F-1492R and sequenced as described by Huang et al. [55]. This 16S rRNA gene sequence was then compared with those found on the EzBioCloud server [57]. A phylogenetic tree was subsequently constructed with MEGA 7 using the maximum likelihood method [58]. The sequence was deposited in the GenBank database under the accession number MG839276.

### 3.3. Selenite Sensitivity Tests

In order to obtain the minimum inhibitory concentration (MIC) of SeO_3_^2−^, the Se03 isolate was precultured in YEP medium at 30 °C in a rotary shaker incubator (180 rpm) for 18 h (the stationary growth phase). The cells were then harvested (centrifuge at 5000× *g* for 10 min) and resuspended in fresh YEP medium. Finally, the cells were challenged (1%, *v*/*v*) in a test tube containing 5 mL of fresh YEP medium to which various concentrations of SeO_3_^2−^ (0–300 mM) had been added. The first concentrations used were 0.5, 1, 3, 5 and 10 mmol/L. Between 10 and 300 mmol/L, the SeO_3_^2−^ concentration was increased in 10 mM increments. After 24 h of incubation at 30 °C, aliquots (100 μL) of the cells in each treatment were spotted onto YEP agar plates and incubated for a further 24 h at 30 °C to determine the concentrations of SeO_3_^2−^ that inhibited bacterial growth.

### 3.4. Evaluation of the Reduction Efficiency of Se03

The reduction efficiency of the Se03 isolate was investigated by adding it to a 500 mL flask containing 200 mL of YEP medium and Na2SeO3 (1 mM or 5 mM) and culturing it at 30 °C in a rotary shaker incubator (180 rpm).

#### 3.4.1. Evaluation of Bacterial Growth Dynamics after Exposure to SeO_3_^2−^

The growth dynamics of the Se03 isolate was investigated by sampling 10 mL of Se03 culture every 6 h from each flask to determine the SeO_3_^2−^ reduction efficiency and Se^0^ levels. At the same time, 100 μL of Se03 culture was sampled every 6 h, serially diluted with sterile ddH_2_O, spotted onto YEP agar plates, and incubated at 30 °C for 24 h. The number of growing cells was counted and reported as the average (*n* = 3) and standard deviation of colony forming units per milliliter (CFU/mL).

#### 3.4.2. Assessment of SeO_3_^2−^ Bio-Reduction Efficiency

Inductively coupled plasma optical emission spectrometry (ICP-OES) (Thermo Fischer Scientific, Waltham, MA, USA) was employed to determine the selenite concentration in each sample as described by Nawaz et al. [59] with slight modifications. Briefly, 10 mL of bacterial culture was sampled every 6 h from each culture, followed by centrifugation at 12,000× *g* for 20 min. After centrifugation, the bacterial cells and elemental selenium were collected as a pellet for subsequent Se^0^ content analysis. The supernatant was passed through a 0.22 μm filter. After that, 300 μL of the supernatant was mixed with 3 mL of HNO_3_, left overnight, and passed through the 0.22 μm filter again. The samples were then diluted to the appropriate selenium concentration and subjected to ICP-OES analysis.

#### 3.4.3. Se^0^ Content Analysis

Se^0^ concentration was determined using the spectrophotometric method described by Khoei et al. [10] with minor modifications. First, the intensity of red Se^0^ produced by reducing selenite solutions of 1–10 μmol with 25 μmol HN_2_OH·HCl was measured at a wavelength of 490 nm to establish a calibration curve. The pellets from the previous centrifugation step were first washed three times with 10 mL of 1 M NaCl to remove selenite contamination, followed by sonication as described by Khoei et al. [10]. After sonication, the pellets were washed twice with 10 mL of 1 M NaCl and dissolved in 10 mL of 1 M Na_2_S. Finally, the samples were centrifuged at 8000× *g* for 20 min to separate the cells, and the absorption of the supernatant (the reddish solution) was measured using spectrophotometry at 490 nm.

### 3.5. Location of SeNPs within the Bacterial Cells

The location of SeNPs within the bacterial cells was determined using TEM and SEM analysis of the bacterial cultures grown either in the YEP medium only (control) or in the YEP medium to which 5.0 mM Na_2_SeO_3_ had been added.

#### 3.5.1. TEM Analysis

The bacterial cultures grown in both the control and in the YEP/Na_2_SeO_3_ mixture were collected after 36 h of incubation at 30 °C. The cells were harvested using centrifugation at 5000× *g* for 6 min and fixed with 2% glutaraldehyde in 0.1 M phosphate-buffered saline (PBS, pH 7.4) for 2 h. The cells then underwent post-fixation treatment in 1% osmium tetroxide for 2 h. The samples were subsequently processed using standard procedures and mounted on copper grids. The analysis was conducted at 80 kV with a Hitachi HT-7700 (Tokyo, Japan) transmission electron microscope.

#### 3.5.2. SEM Analysis

The bacterial cultures grown in the control and in the YEP/Na_2_SeO_3_ mixture were collected after 36 h incubation at 30 °C. The cells were harvested using centrifugation at 5000× *g* for 5 min and fixed overnight in 2.5% glutaraldehyde at 4 °C. Following this, the cells were washed twice in PBS (0.1 M, pH 7.4) and dehydrated using an ethanol series (30%, 50%, 70%, 80%, 90%, and 100%). Finally, they were dried using the critical point drying method with CO_2_ and analyzed with an S4800 (Tokyo, Japan) scanning electron microscope.

### 3.6. Analysis of SeNPs

#### 3.6.1. Preparation of Biogenic SeNPs

The Se03 isolate was cultured in 500-mL flasks containing 200 mL of YEP medium and 5 mM Na_2_SeO_3_ at 30 °C in a rotary shaker (180 rpm) for 36 h. After incubation, the bacterial culture was centrifuged at 10,000× *g* at 4 °C for 10 min. The pellet was collected and rinsed twice with 0.9% NaCl. After this, the pellet was resuspended in 20 mL of Tris-Cl buffer (50 mM, pH 8.2) and disrupted by ultra-sonication on ice (ten cycles of 40 s of sonication with 40 s of rest). After sonication, the suspension was centrifuged at 12,000× *g* for 40 min. The pellet was discarded while the supernatant was centrifuged at 40,000× *g* at 4 °C for 40 min to harvest the nanoparticles. Finally, the subsequent pellet containing the SeNPs was washed twice with water and then resuspended in deionized water.

#### 3.6.2. Dynamic Light Scattering (DLS) Analysis

DLS analysis was conducted using a Zen 3600 Zetasizer Nano ZS from Malvern Instruments Ltd. (Worcestershire, UK) equipped with a 633-nm helium-neon laser light source (4.0 mW). Before the measurement, the SeNPs suspensions were diluted to 1:10 to 1:20 to meet the optical requirements of the instrument, and the mean size distribution of the selenium particles was determined as described by Lampis et al. [29].

#### 3.6.3. Scanning Electron Microscopy and Electron Dispersive Spectrometry (SEM-EDS) Analysis

The morphology and constituent elements of the purified SeNPs were analyzed using SEM-EDS analysis. For this purpose, the SeNPs suspensions were first dried with a vacuum freeze dryer (SJIA-10N, Ningbo YinZhou Sjia Lab Equipment Co., Ltd., YinZhou, China) and the selenium particles were then analyzed with a scanning electron microscope (Hitachi S4800, Tokyo, Japan) with energy-dispersive X-ray spectra (SEM-EDS).

#### 3.6.4. Fourier Transform Infrared (FTIR) Spectroscopy Analysis

For the FTIR analysis, the SeNP suspensions were first dried with a vacuum freeze dryer (SJIA-10N, Ningbo YinZhou Sjia Lab Equipment Co., Ltd., YinZhou, China). Mid-infrared spectroscopy (4000–400 cm^−1^) was applied in transmission mode employing a Nicolet IS10 spectrometer (Thermo Fischer Scientific, Waltham, MA, USA).

### 3.7. Biocatalytic Selenite Reduction Assays

To demonstrate which fractions of the cell culture (the cytoplasm, periplasm, membrane, and exopolysaccharide [EPS] fractions, and the culture supernatant) were responsible for selenite reduction and SeNPs formation, and to clarify the possible reduction mechanisms, the biocatalytic reduction of selenite was assessed with the different fractions of Se03.

#### 3.7.1. Protein Extraction

To extract the cell protein fractions, Se03 was cultured in 1-L flasks containing 400 mL of YEP medium at 30 °C in a rotary shaker (180 rpm) for 20 h. The bacterial culture was then centrifuged at 10,000× *g* at 4 °C for 10 min to collect the cell pellet. Following this, the pellet was washed twice with 0.9% NaCl and pelleted again. The periplasmic, membrane, fractions and soluble cytoplasmic fractions were then extracted following the process described by Khoei et al. [10]. In addition, the protein content of the individual fractions was determined using Bradford assays with bovine serum albumin as the standard.

#### 3.7.2. EPS Extraction

To extract the EPS fraction, 100 mL of the Se03 culture incubated in YEP at 30 °C for 5 d was centrifuged at 12,000× *g* at 4 °C for 30 min and the pellet was discarded. The supernatant was then passed through a 0.22 μm filter and precipitated with an equal volume of cold ethanol at −20 °C overnight. Following this, the pellet containing the EPS fraction was harvested using centrifugation at 12,000× *g* for 30 min at 4 °C and dissolved in sterile ddH_2_O.

#### 3.7.3. Supernatant Preparation

Stationary phase cultures (incubated for 24 h) of Se03 in YEP were centrifuged at 10,000× *g* for 5 min at 4 °C. The supernatant was passed through a 0.22 μm filter and collected.

#### 3.7.4. Selenite Reducing Reduction Activity Assays

The selenite reduction activity of the individual fractions of the bacterial cultures was assessed as described by Khoei et al. [10] with minor modifications. Generally, the appearance of a red color in the microplate wells indicated the production of elemental selenium.

The activity assays were performed in 96-well plates. First, 100 μL of the protein (2 mg/mL), EPS, or supernatant samples was carefully transferred into the 96-well plate. Subsequently, 88 μL of McIlvaine buffer, 10 μL of sodium selenite solution (a final concentration of 5.0 mM), and 2 μL of NADH (electron donor, final concentration 2.0 mM) were added to each well to obtain a final volume of 200 μL. Afterwards, the mixture was incubated at 30 °C for at least 72 h. The experiment was repeated with NADPH as an alternative electron donor). Three negative controls were set: Without selenite, without an electron donor, and without the cell protein/EPS/supernatant. The presence of red in the wells, indicating the production of elemental selenium, was interpreted as a positive result.

### 3.8. Real-Time Quantitative PCR (qPCR)

To determine the proteins that might be involved in the reduction of SeO_3_^2−^ to SeNPs, the mRNA expression of various enzymes, including sulfite reductase (*cys*I) and flavoprotein sulfite reductase (*BV899_18870*), in response to selenite treatment were assessed using qPCR. The gene-specific primers are presented in Table 2 with the 16S rRNA gene sequence as a reference.

Bacterial cultures grown in YEP medium (control) or in YEP medium to which 5.0 mM Na_2_SeO_3_ had been added were collected after 24 h of incubation at 30 °C. After incubation, total bacterial RNA was extracted from 2 mL of bacterial culture using an E.Z.N.A. bacterial RNA kit (Omega Bio-tek, USA) following the manufacturer’s protocol, and the RNA concentration was determined using a ScanDrop spectrophotometer (Analytik Jena AG, Jena, Germany). cDNA was then synthesized using TransScript One-Step gDNA Removal and cDNA Synthesis SuperMix (TransGen Biotech, Peking, China). Afterwards, the cDNA was subjected to qPCR analysis using a QuantiFast SYBR Green PCR kit (Qiagen, Germany) on a Roche LC96 real-time PCR machine (Roche Diagnostics, Indianapolis, IN, USA). The qPCR process was as follows: One cycle at 95 °C for 10 min, followed by 45 cycles at 95 °C for 10 s, 55 °C for 20 s, and 72 °C for 20 s. Melting curve analysis was performed moving from 60 °C to 95 °C by 0.5 °C every 2 s. Variation in gene expression was evaluated in terms of the change with respect to the untreated cells (control) using the 2^−∆∆*C*t^ method. Each sample was tested three times and three independent tests were conducted.

### 3.9. Statistical Analysis

Normality of continuous data was determined by Shapiro-Wilk normality test. One-way analysis of variance (ANOVA) at a 95% confidence level followed by Tukey’s test was used to determine the statistical significance of pairwise comparisons. All analyses were conducted in triplicate, and all values presented are the average of three independent sets and expressed as the mean ± standard deviation (S.D.). The level of significance was set at *p* < 0.05.

## 4. Conclusions

In summary, the bacterium strain *A. faecalis* Se03, which has a high selenite tolerance and a strong reduction ability, was isolated from the gut of *M. alternatus* larvae. To the best of our knowledge, this is the first study to demonstrate that *A. faecalis* is capable of efficiently transforming toxic SeO_3_^2−^ to Se^0^ and generating SeNPs in a liquid growth medium under aerobic growth conditions. The in vitro synthesis experimental results indicated that SeNP formation can be tentatively attributed to cytoplasmic enzymatic activation mediated by electron donors (NADPH or NADH). However, intracellular elemental selenium was also observed, even though the SeNPs were primarily located in the extracellular space. Therefore, it is likely that the biogenic SeNPs were released outside the bacterial cells as a consequence of a secretory process or cell lysis. Further studies are needed in order to clarify the specific mechanisms of SeNP synthesis and how these particles are transported out of cells.

## Figures and Tables

**Table 1:** Physiological and biochemical characteristic of the bacterial isolate Se03.

Characteristic	Result	Characteristic	Result
Gram-staining	−	Assimilation of	
Nitrite reduction	+	Glycine	−
Motility	+	Lactose	+
Oxidase	+	Maltose	+
Catalase	+	d-fructose	+
Indole test	−	d-Raffinose	+
Nitrate reduction	+	Cellobiose	+
Urease	+	Rhamnose	+
Hydrolysis of		Sucrose	+
Starch	−	d-xylose	−
Casein	−	Arginine dihydrolase	+
Gelatin	−	Growth in 5% NaCl	−
Growth at 5 °C	+	Growth in 10% NaCl	−
Growth at 42 °C	+		

Growth/Posetive result(+), No growth/Negtive result(−).

**Table 2:** Primers for targeting genes.

Target Gene	Primer Sequence	Product Size (bp)
Glutathione synthetase (A0A1Y1PZ31)	Forward: 5′-CCCAAAGTCGGGTTCGT-3′ Reverse: 5′-CAAGTGCGTGGAATAGGAGTA-3′	181
Sulfite reductase (A0A1Y1PQF9)	Forward: 5′-TCAAGAGTGGGCTGACAAGA-3′ Reverse: 5′-CACATCATTCAAGGGAGGC-3′	186
Sulfate transporter subunit (A0A1Y1PY15)	Forward: 5′-CAAAGAGCAAACGGGTGA-3′ Reverse: 5′-ACAATCGTGGAGGTGTAAGG-3′	209
Flavoprotein sulfite reductase (A0A1Y1PQH1)	Forward: 5′-TTCCTGCCGCCCAATCTA-3′ Reverse: 5′-TGCCTTCCAGCGACAACTC-3′	161
Thioredoxin reductase (A0A1Y1PXH0)	Forward: 5′-CAGGCTGCGGCGATTCAA-3′ Reverse: 5′-TCTGGGCGTCCTCGGTCAA-3′	249
Peroxiredoxin (A0A1Y1PUT6)	Forward: 5′-CATTGAAACCGCCACAGA-3′ Reverse: 5′-CGCCCATTACGAAAGGAT-3′	223
Superoxide dismutase (A0A1Y1PZA2)	Forward: 5′-CCCGATGCCGATAGCACCCT-3′ Reverse: 5′-ACTGAGCCAAGCCCAGCCAC-3′	134
16S r RNA	Forward: 5′-AGAGTTTGATCCTGGCTCAG-3′ Reverse: 5′-CTGCTGCCTCCCGTAGGAGT-3′	330

## Supplementary Materials

Supplementary materials can be found at http://www.mdpi.com/1422-0067/19/9/2799/s1.

## Abbreviations

SeNPs	Se nanoparticles
Se^0^	Elemental Selenium
TEM	Transmission electron microscopy
SEM	Scanning electron microscopy
EDX	Energy dispersive X-ray analysis
MIC	Minimum inhibitory concentration
NADH	Nicotinamide adenine dinucleotide
NADPH	Nicotinamide adenine dinucleotide phosphate
qPCR	Real-time quantitative PCR
