# Correction: Wang, Y.T., et al. Selenite Reduction and the Biogenesis of Selenium Nanoparticles by *Alcaligenes faecalis* Se03 Isolated from the Gut of *Monochamus alternatus* (Coleoptera: Cerambycidae). *Int. J. Mol. Sci.* 2018, *19*, 2799

**DOI:** 10.3390/ijms21041294

**Published:** 2020-02-14

**Authors:** Yuting Wang, Xian Shu, Qing Zhou, Tao Fan, Taichu Wang, Xue Chen, Minghao Li, Yuhan Ma, Jun Ni, Jinyan Hou, Weiwei Zhao, Ruixue Li, Shengwei Huang, Lifang Wu

**Affiliations:** 1 Key Laboratory of High Magnetic Field and Ion Beam Physical Biology, Hefei Institutes of Physical Science, Chinese Academy of Sciences, Hefei 230031, China; 2 School of Life Sciences, University of Science and Technology of China, Hefei 230026, China; 3 The Sericultural Research Institute, Anhui Academy of Agricultural Science, Hefei 230061, China; 4 Key Laboratory of Environmental Toxicology and Pollution Control Technology of Anhui Province, Hefei Institutes of Physical Science, Chinese Academy of Sciences, Hefei 230031, China

The authors wish to make the following corrections to this paper [1]:

## 1. Change in Main Body Paragraphs

The authors are sorry to report that some of the FTIR data reported in their recently published paper [1] were incorrect. We have recently been made aware by Prof. Alexander A. Kamnev (Institute of Biochemistry and Physiology of Plants and Microorganisms, Russian Academy of Sciences, Saratov) that the FTIR spectrum was presented and interpreted erroneously because it was illustrated with the vertical (intensity) axis “Transmittance“ (with peak maxima directed downwards) while the spectrum itself was plotted as if with the vertical (intensity) axis “Absorbance” (with real absorption peaks directed upwards). Consequently, the authors wish to make, at this time, the following corrections to the paper:

Abstract:

Replace the sentence “Finally, using Fourier-transform infrared spectrometry, protein and lipid residues were detected on the surface of the biogenic SeNPs” by “Finally, using Fourier transform infrared spectroscopy, protein and carbohydrate residues were detected on the surface of the biogenic SeNPs”.

2.4.2. FTIR analysis

The Fourier transform infrared (FTIR) spectrum of the SeNPs is presented in Figure 7. The bands observed at 3270 cm^−1^, 3184 cm^−1^, and at 3070 cm^−1^ may be assigned to different N−H stretching vibrations and amide A of proteins, respectively. A number of typical C−H stretching bands can also be observed at 2955, 2923, 2872, and 2850 cm^−1^ (aliphatic groups). The band at 1660 cm^−1^ with the accompanying less-intensive bands at 1528 and 1230 cm^−1^ represent the amide I, amide II, and amide III modes, respectively, typical of proteins. The weaker band at 1456 cm^−1^ corresponds to the CH_2_ scissoring mode. Note that the band at 1400 cm^−1^ may be assigned to the symmetric stretching vibrations of carboxylate (COO^−^), while its asymmetric counterpart may be seen as a peak around 1620 cm^−1^ (largely overlapped by the amide I band). The bands at 1164 and 1070 cm^−1^ are typical of C–O–C and C–O vibrations in carbohydrates, which may represent the presence of polysaccharides in the capping layer of the SeNPs. Thus, the FTIR analysis clearly demonstrated that organic residues such as carbohydrates and proteins were present on the surface of the SeNPs produced by *A. faecalis* Se03. Previous studies on *Thauera selenatis* [38] and *S. maltophilia* SeITE02 [29] have identified the functional groups of the bacterial biomolecules involved in the selenite reduction and the stabilization (capping) process for synthesized SeNPs. Therefore, our findings not only indicate bacterial protein-mediated selenite reduction but also corroborate the synthesis and stabilization of SeNPs by the proteins present in the bacteria [38].

## 2. Change in Figure

The author wishes to make the following correction to this paper [1]. Due to mislabeling, replace:

**Figure 7:**
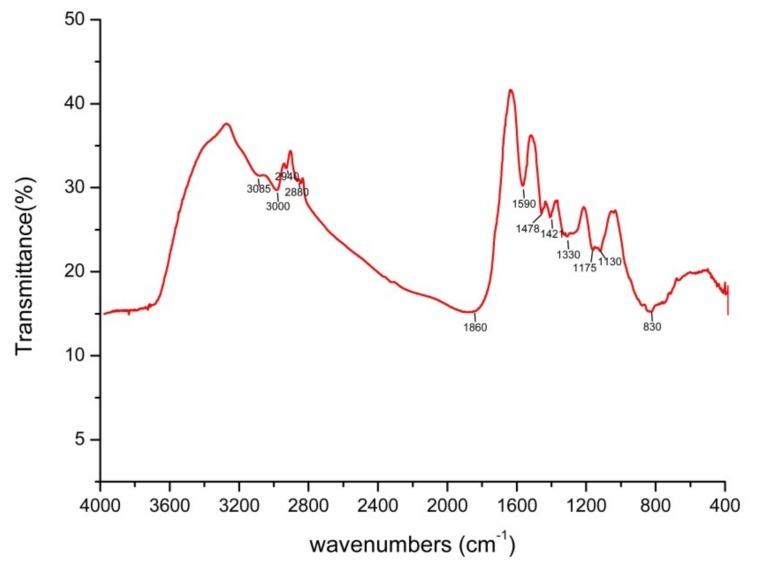
The FTIR spectrum of Bio-SeNPs registered in the 4000–400 cm^−1^ infrared regions.

with the corrected Figure 7 (Figure 1):

**Figure 1:**
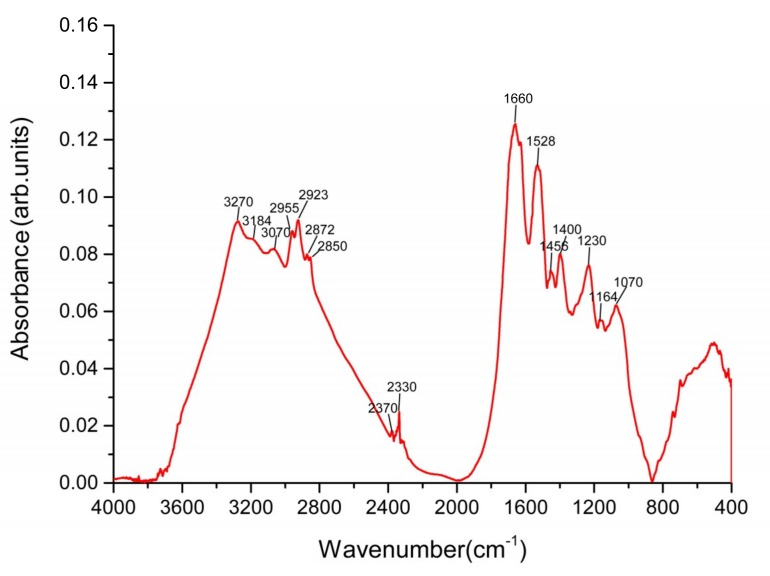
The FTIR spectrum of Bio-SeNPs registered in the 4000–400 cm^−1^ infrared regions.

These changes have no material impact on the conclusions of our paper. We apologize to our readers. The authors would like to apologize for any inconvenience caused to the readers by these changes.

## References

[1] Huang S., Wu L., Wang Y., Shu X., Zhou Q., Fan T., Wang T., Chen X., Li M., Ma Y. (2018). Selenite Reduction and the Biogenesis of Selenium Nanoparticles by *Alcaligenes faecalis* Se03 Isolated from the Gut of *Monochamus alternatus* (Coleoptera: Cerambycidae). Int. J. Mol. Sci..

