# Supplementary material for: Selenite Reduction and the Biogenesis of Selenium Nanoparticles by Alcaligenes faecalis Se03 Isolated from the Gut of Monochamus alternatus (Coleoptera: Cerambycidae)
Source: Int J Mol Sci. 2018 Sep 17;19(9):2799. doi: 10.3390/ijms19092799 (PMC6164237; doi:10.3390/ijms19092799)
Supplement: Supplementary file 1 [file ijms-19-02799-s001.pdf]

Table S1-1 Shapiro-Wilk test results of bacterial growth of Se03 (without SeO_3^{2-})*

Time courses	Mean of 3 Replicates (Log_{10} CFU/mL)	SD	W Statistics	*P*-value	Level of significance	Conclusion
0	6.10333	0.03215	0.95107	0.57402	0.05	Accept
6	6.34667	0.08737	0.94651	0.08737	0.05	Accept
12	6.73000	0.08544	0.98973	0.08544	0.05	Accept
18	7.31333	0.05686	0.93557	0.05686	0.05	Accept
24	7.84667	0.08021	0.99482	0.08021	0.05	Accept
30	7.97667	0.18771	0.97138	0.18771	0.05	Accept
36	8.02667	0.10693	0.96429	0.10693	0.05	Accept
42	7.87333	0.11015	0.99725	0.11015	0.05	Accept
48	7.81333	0.11676	0.97005	0.11676	0.05	Accept

* Shapiro-Wilk test: H0:Data are normally distributed; HA: Data are not normally distributed

Table S1-2 Shapiro-Wilk test results of bacterial growth of Se03 (with 1 mM SeO_3^{2-})*

Time courses	Mean of 3 Replicates (Log_{10} CFU/mL)	SD	W Statistics	*P*-value	Level of significance	Conclusion
0	6.06011	0.0755	0.98684	0.78044	0.05	Accept
6	6.37667	0.07572	0.85465	0.25297	0.05	Accept
12	6.54333	0.06429	0.87097	0.29827	0.05	Accept
18	7.23333	0.06807	0.91187	0.42435	0.05	Accept
24	7.88010	0.09539	0.99176	0.82638	0.05	Accept
30	7.89333	0.06506	0.99803	0.91523	0.05	Accept
36	7.92667	0.08505	0.88479	0.33861	0.05	Accept
42	7.75667	0.06807	0.91187	0.42435	0.05	Accept
48	7.64333	0.05508	0.82418	0.17362	0.05	Accept

* Shapiro-Wilk test: H0:Data are normally distributed; HA: Data are not normally distributed

Table S1-3 Shapiro-Wilk test results of bacterial growth of Se03 (with 5 mM SeO_3^{2-})*

Time courses	Mean of 3 Replicates (Log_{10} CFU/mL)	SD	W Statistics	*P*-value	Level of significance	Conclusion
0	6.05667	0.07767	0.93232	0.49736	0.05	Accept
6	6.29100	0.07211	0.94231	0.53674	0.05	Accept
12	6.54333	0.05508	0.82418	0.17362	0.05	Accept
18	7.35667	0.10017	0.8995	0.38392	0.05	Accept
24	7.77333	0.08505	0.99885	0.93516	0.05	Accept
30	7.84000	0.09539	0.99176	0.82638	0.05	Accept
36	7.77667	0.07638	0.96429	0.63689	0.05	Accept
42	7.73000	0.09165	0.96429	0.63689	0.05	Accept
48	7.63333	0.10786	0.8596	0.26647	0.05	Accept

* Shapiro-Wilk test: H0:Data are normally distributed; HA: Data are not normally distributed

Table S1-4 Shapiro-Wilk test results of SeO_3^{2-} concentration in Se03 culture at different culture times (1 mM SeO_3^{2-})*

Time courses	Mean of 3 Replicates (mM)	SD	W Statistics	*P*-value	Level of significance	Conclusion
0	0.99010	0.01000	1.00000	1.00000	0.05	Accept
6	0.95400	0.00889	0.91456	0.43346	0.05	Accept
12	0.89333	0.01762	0.98684	0.78044	0.05	Accept
18	0.52600	0.01136	0.85465	0.25297	0.05	Accept
24	0.07480	0.00632	0.99324	0.84283	0.05	Accept
30	0.01247	0.0015	0.99852	0.92652	0.05	Accept
36	0.00467	0.00306	0.96429	0.63689	0.05	Accept
42	0.00283	0.00229	0.92308	0.46326	0.05	Accept
48	0.00179	0.0025	0.99838	0.92311	0.05	Accept

* Shapiro-Wilk test: H0:Data are normally distributed; HA: Data are not normally distributed

Table S1-5 Shapiro-Wilk test results of SeO_3^{2-} concentration in Se03 culture at different culture times (5 mM SeO_3^{2-})*

Time courses	Mean of 3 Replicates (mM)	SD	W Statistics	*P*-value	Level of significance	Conclusion
0	4.98333	0.01528	0.96429	0.63689	0.05	Accept
6	4.91000	0.06000	1.0000	1.00000	0.05	Accept
12	4.81267	0.05605	0.9983	0.92128	0.05	Accept
18	4.53400	0.17868	0.79683	0.10694	0.05	Accept
24	3.98167	0.17614	0.9815	0.73939	0.05	Accept
30	2.80967	0.09501	0.99985	0.97679	0.05	Accept
36	0.82437	0.01240	0.94469	0.54660	0.05	Accept
42	0.15067	0.01528	0.96429	0.63689	0.05	Accept
48	0.00094	0.00106	0.77803	0.06307	0.05	Accept

* Shapiro-Wilk test: H0:Data are normally distributed; HA: Data are not normally distributed

Table S1-6 Shapiro-Wilk test results of Se^0 concentration in Se03 culture at different culture times (1 mM SeO_3^{2-})*

Time courses	Mean of 3 Replicates (mM)	SD	W Statistics	*P*-value	Level of significance	Conclusion
0	0.00910	0.0004	1.00000	1.00000	0.05	Accept
6	0.02110	0.0030	1.00000	1.00000	0.05	Accept
12	0.05120	0.00917	0.96429	0.63689	0.05	Accept
18	0.21333	0.01662	0.98522	0.76726	0.05	Accept
24	0.54533	0.04477	0.98798	0.79023	0.05	Accept
30	0.73067	0.02082	0.92308	0.46326	0.05	Accept
36	0.87533	0.01716	0.98188	0.74213	0.05	Accept
42	0.91200	0.01825	0.81757	0.15717	0.05	Accept
48	0.93710	0.02402	0.91681	0.44121	0.05	Accept

* Shapiro-Wilk test: H0:Data are normally distributed; HA: Data are not normally distributed

Table S1-7 Shapiro-Wilk test results of Se^0 concentration in Se03 culture at different culture times (5 mM SeO_3^{2-})*

Time courses	Mean of 3 Replicates (mM)	SD	W Statistics	*P*-value	Level of significance	Conclusion
0	0.00014	0.00068	0.81757	0.15717	0.05	Accept
6	0.05067	0.00709	0.97351	0.68777	0.05	Accept
12	0.10200	0.01758	0.88107	0.32755	0.05	Accept
18	0.32667	0.02892	0.90424	0.39914	0.05	Accept
24	0.72733	0.04041	0.97959	0.72623	0.05	Accept
30	1.85233	0.01501	0.99852	0.92652	0.05	Accept
36	3.42167	0.12846	0.77969	0.06687	0.05	Accept
42	4.40800	0.19010	1.00000	1.00000	0.05	Accept
48	4.52500	0.07622	0.9887	0.79658	0.05	Accept

* Shapiro-Wilk test: H0:Data are normally distributed; HA: Data are not normally distributed

Table S1-8 Shapiro-Wilk test results of expression levels of selected genes assessed using real-time PCR (fold changes)*

genes	Mean of 3 Replicates (fold changes)	SD	W Statistics	*P*-value	Level of significance	Conclusion
*gsh*B	1.03513	0.13191	0.88351	0.3348	0.05	Accept
BV899_18870	1.08669	0.05032	0.96086	0.61966	0.05	Accept
*Cys*I	4.22199	0.16337	0.99745	0.90348	0.05	Accept
BV899_03375	2.23210	0.04459	0.89218	0.36101	0.05	Accept
BV899_02360	4.77585	0.14891	1.00000	0.99600	0.05	Accept
BV899_10955	1.94098	0.10633	0.9906	0.81454	0.05	Accept
BV899_06125	9.62880	1.18768	0.92511	0.4706	0.05	Accept

* Shapiro-Wilk test: H0:Data are normally distributed; HA: Data are not normally distributed; *gsh*B: glutathione synthetase, *BV899_18870*: flavoprotein sulfite reductase; *Cys*I: sulfite reductase , *BV899_03375*:sulfate transporter subunit; *BV899_02360*: thioredoxin reductase, *BV899_10955*:peroxiredoxin, *BV899_06125*:superoxide dismutase
